# Dementia in a resource‐constrained sub‐Saharan African setting: A comprehensive retrospective analysis of prevalence, risk factors, and management at the only neuropsychiatric facility in Northeastern Nigeria

**DOI:** 10.1002/alz.14538

**Published:** 2025-03-20

**Authors:** Ibrahim Abdu Wakawa, Umar Baba Musami, Suleiman Hamidu Kwairanga, Placidus Nwankuba Ogualili, Mohammed Yusuf Mahmood, Muhammad Abba Fugu, Mohammed Mala Gimba, Muktar Mohammed Allamin, Zaharadeen Umar Abbas, Muhammad Kawu Sunkani, Zainab Bukar Yaganami, Fatima Mustapha Kadau, Nasir Muhammad Sani, Peter Danmallam, Luka Nanjul, Larema Babazau, Zaid Muhammad, Baba Waru Goni, Babagana Kundi Machina, Celeste M. Karch, Chinedu Udeh‐Momoh, Thomas K. Karikari, Chiadi U. Onyike, Mahmoud Bukar Maina

**Affiliations:** ^1^ Department of Medical Services Federal Neuropsychiatric Hospital Maiduguri Borno Nigeria; ^2^ Department of Mental Health College of Medical Sciences University of Maiduguri Maiduguri Borno Nigeria; ^3^ Department of Human Anatomy Faculty of Basic and Allied Medical Sciences College of Medical Science Gombe State University Tudun Wada Gombe Nigeria; ^4^ Biomedical Science Research and Training Centre Damaturu Damaturu Yobe Nigeria; ^5^ Geriatric Unit Department of Medical Services Federal Neuropsychiatric Hospital Maiduguri Borno Nigeria; ^6^ Department of Health Information Federal Neuropsychiatric Hospital Maiduguri Borno Nigeria; ^7^ Department of Medical Laboratory Services Yobe State University Teaching Hospital Damaturu Nigeria; ^8^ Department of Human Physiology College of Medical Sciences Yobe State University, Damaturu Damaturu Yobe Nigeria; ^9^ Department of Medicine Yobe State University Teaching Hospital Damaturu Nigeria; ^10^ Department of Medicine University of Maiduguri University of Maiduguri Teaching Hospital Maiduguri Borno Nigeria; ^11^ Department of Psychiatry Yobe State Specialist Hospital Damaturu Yobe Nigeria; ^12^ Department of Psychiatry Yobe State University Teaching Hospital Damaturu Nigeria; ^13^ Department of Psychiatry Washington University School of Medicine St. Louis Missouri USA; ^14^ Division of Clinical Geriatrics Center for Alzheimer Research, Karolinska Institute Stockholm Sweden; ^15^ Sheffield Institute for Translational Neuroscience (SITraN) University of Sheffield Sheffield UK; ^16^ School of Public Health Sciences Wake Forest University School of Medicine North Carolina USA; ^17^ Brain and Mind Institute Aga Khan University Nairobi Kenya; ^18^ Department of Psychiatry and Neurochemistry Institute of Neuroscience and Physiology University of Gothenburg Gothenburg Sweden; ^19^ Department of Psychiatry School of Medicine University of Pittsburgh Pittsburgh Pennsylvania USA; ^20^ Department of Psychiatry and Behavioural Sciences Johns Hopkins University School of Medicine Baltimore Maryland USA; ^21^ Sussex Neuroscience School of Life Sciences University of Sussex Brighton UK

**Keywords:** Alzheimer's disease, dementia, dementia management, Northeastern Nigeria, resource‐constrained settings, sub‐Saharan Africa, vascular dementia

## Abstract

**INTRODUCTION:**

Dementia prevalence is increasing in sub‐Saharan Africa, potentially due to population growth and aging. Resource‐constrained settings such as Northeastern Nigeria face challenges in dementia management.

**METHODS:**

We assessed dementia burden and management at the Federal Neuropsychiatric Hospital Maiduguri, the only neuropsychiatric facility in Northeastern Nigeria. This retrospective analysis included patient records from 1999 to 2023 for individuals 60 year of age and older with a dementia diagnosis.

**RESULTS:**

Of the 1216 cases reported, Alzheimer's disease (60.5%) was the most common subtype, followed by vascular dementia (24.5%). Hypertension (41.6%) was the most frequent comorbidity. Memory loss was present in all cases, whereas behavioral symptoms like agitation presented in some cases. Treatments included cognitive enhancers (donepezil), supplements (gingko biloba), and non‐drug therapies (psychoeducation).

**DISCUSSION:**

The increasing burden of dementia at this sole facility highlights the urgent need for targeted interventions and further research to understand the underlying factors contributing to dementia in this population.

**Highlights:**

- Dementia trends and management in a neuropsychiatric facility serving over 26 million people in Northeastern Nigeria.
- Alzheimer's disease accounted for 60.5% of the dementia cases reported, with hypertension as the leading comorbidity.
- There is an urgent need for improved diagnostic tools and health care infrastructure to address dementia in resource‐constrained settings.
- The findings lay the foundation for developing a dementia cohort as part of the Northern Nigeria Dementia Research Group.

## 1 BACKGROUND

Sub‐Saharan Africa is one of the regions most affected by the increase in the population of older adults 60 years of age and above.^1^, ^2^, ^3^ From ≈24 million in 1980 to 74 million by 2020, the total number of older Africans is projected to triple between 2020 and 2050.^4^ Nigeria, the most populous country in Africa, has the highest number of older adults and is nineteenth worldwide.^4^ The consequences in terms of increased occurrence of both preclinical and symptomatic dementia are grave.

Dementia is typically characterized by impairment in multiple domains such as memory loss, executive dysfunction, impairments in basic and instrumental activities of daily living (IADLs), as well as behavioural and psychological symptoms of dementia (BPSD).^5^, ^6^, ^7^, ^8^, ^9^ In addition, psychiatric comorbidities and other medical disorders are common.^10^ The impact is felt not only by the patient but also by the primary caregivers and clinicians.^11^

The burden of dementia is disproportionately higher in low‐ and middle‐income countries (LIMCs), particularly in sub‐Saharan Africa.^1^ Late presentation and diagnosis, limited access to specialist care, inadequate health care infrastructure and resources, and a lack of culturally appropriate interventions represent unique challenges in the management of dementia in these settings.^12^, ^13^

In sub‐Saharan Africa, there is generally a lack of peer‐reviewed information on clinical dementia due to challenges in diagnosis. Biomarkers are often unavailable, and neuroimaging techniques are rarely used.^1^ Even in cases where neuroimaging is accessible, it is limited to structural imaging, such as magnetic resonance imaging (MRI). At the same time, more advanced modalities like positron emission tomography (PET) scanners are mostly unavailable.

Two seminal studies conducted on dementia in Nigeria were the Indianapolis‐Ibadan Dementia Research Project and the Ibadan Study of Aging, which were carried out over three and two decades ago, respectively, in southwestern Nigeria.^14^, ^15^, ^16^ Both were community studies that assessed the prevalence and risk factors of dementia in elderly adults. The outcomes of both studies revealed some degrees of variation when compared to findings from Western countries, particularly those from Europe and North America. Although environmental factors might influence these differences, genetic factors, such as variations in the apolipoprotein E (*APOE*) gene and haplotype, likely play a significant role.^17^, ^18^, ^19^ In addition, other hospital‐based studies have described dementia patterns in Nigeria using clinical diagnostic criteria and validated bedside instruments for cognitive assessment, such as the Mini‐Mental State Examination (MMSE) and the Montreal Cognitive Assessment (MoCA).^20^, ^21^, ^22^, ^23^ The diagnosis of other comorbidities is often clinical and depends on the examining clinician. There is a high tendency that psychiatric comorbidities are likely to be underdiagnosed or not detected completely. Furthermore, the lack of diagnostic biomarkers and neuroimaging techniques limits the validity of the diagnosis made. In northern Nigeria, a community‐based study used the International Classification of Diseases, 10th Revision (ICD‐10), and the Diagnostic and Statistical Manual of Mental Disorders, Fourth Edition (DSM‐IV), to diagnose dementia. The study reported a dementia prevalence of 2.79%, with a 95% confidence interval (CI) of 1% to 4.58%.^24^

This study retrospectively examined the clinical management of dementia in a resource‐constrained, sub‐Saharan African neuropsychiatric hospital, the largest tertiary care facility serving the over 26 million people of Northeastern Nigeria.^25^, ^26^, ^27^ We sought to provide comprehensive insights into the approaches taken in the environment and identify potential areas for improvement in health care delivery. The study aimed to analyze the trends in clinical dementia cases among elderly Nigerians (60 years of age and above) who presented at the Federal Neuropsychiatric Hospital Maiduguri from all states of Nigeria since its inception. In addition, to understand clinical patterns according to the various counties in the home state of Borno where the hospital is located, we determined the number of reported cases by local government area, adjusted for population. We also identified the subtypes of dementia diagnoses in the last 5 years, assessed the prevalence of psychiatric comorbidities, reviewed routine laboratory investigations and reported symptoms, and evaluated the medications used for dementia management in order to enhance the understanding of dementia care and highlight the challenges and opportunities for improving dementia management in our region and in sub‐Saharan Africa.

## 2 METHODS

### 2.1 Study location

This study was carried out at the Psychogeriatric Unit of the Federal Neuropsychiatric Hospital, Maiduguri (FNPHM), Nigeria, a specialized institution established in 1999 to serve as the primary neuropsychiatric referral center for the six states within Nigeria's North‐East geopolitical zone. These states have a combined population exceeding 22 million, based on the estimation from the most recent national census.^25^, ^26^

**RESEARCH IN CONTEXT**

1. **Systematic review**: Previous studies, including the Indianapolis‐Ibadan Dementia Project and the Ibadan Study of Aging, have offered valuable insights into dementia in Southwestern Nigeria. However, there is a notable gap in research from Northern Nigeria—the country's most populous region.
2. **Interpretation**: Our study, based on data from the only neuropsychiatric hospital in Northeastern Nigeria, representing over 26 million people, documented 1216 dementia cases in individuals 60 years of age and older. Alzheimer's disease (60.5%) and vascular dementia (24.5%) were the most common subtypes, with a high prevalence of comorbid hypertension, aligning with global patterns.
3. **Future directions**: To advance dementia research in this underrepresented population, it is essential to explore the genetic, environmental, and lifestyle factors specific to this region and their role in dementia biology. We have established the Northern Nigeria Dementia Research Group to help build a dementia cohort to further our understanding of the disease and enhance regional and global dementia research.

### 2.2 Study participants

The study population comprised elderly patients 60 years of age and above who had been managed within the Psychogeriatric Unit of the Federal Neuro‐Psychiatric Hospital (FNPHM), Nigeria since its inception in 1999. The study participants had a diagnosis of dementia by the World Health Organization's (WHO) Tenth Revision of the International Classification of Desease (ICD‐10) code number F01‐F99.

### 2.3 Study procedure

This was a retrospective observational analysis of data abstracted from the hospital's electronic medical record (EMR) system, which provided detailed patient histories, including demographic information, dementia type, MMSE scores, BPSD, comorbidities, and results from baseline laboratory investigations. The diagnoses of dementia and its subtypes were based on the ICD‐10 and DSM‐5 criteria, ascertained by a consultant neuropsychiatrist, and independently confirmed by another. The MMSE was used as a quantitative tool to assess cognitive function and support clinical diagnoses. Paper records (case notes) were reviewed manually to ensure the completeness and accuracy of the collected data and to collect data in instances where EMR data were incomplete or unavailable. However, some cases could not be used due to the inability to trace records data, ineligibility (such as missing critical content), or cases where the diagnosis was not confirmed by a consultant or was not based on ICD criteria. A data abstraction template was designed and built into a data entry application using Kobo toolbox to streamline the data entry process. This tool was formatted to contain all the necessary sociodemographic and clinical information. A training program was organized for those involved in data entry to ensure uniformity, reliability, and standards. This enabled the study doctors to mine the data from the case notes of the patients and enter them directly into the database. The data extraction was conducted between August 1 and 20, in 2024. Once the data entry was completed, the data set was exported as a Comma Separated Values (CSV) file for analysis.

### 2.4 Data analysis

Descriptive statistics are reported. Data analysis for this study was conducted using Anaconda Navigator and Jupyter Notebook for Python 3.10. Pandas and geopandas were used for data processing and descriptive statistics. Matplotlib and Seaborn's packages were used to plot the stack histograms, geoplots for the choropleth map, and UpSet plot to explore the intersections of symptoms, comorbidities, diagnostic data, and treatments, and the Upset data were used to describe correlations.

### 2.5 Ethical considerations

The study was conducted in full compliance with ethical standards. Ethical approval was obtained from the National Human Research Ethical Review Committee through the institutional review board of the FNPHM. The study adhered to strict confidentiality protocols, ensuring all patient information was anonymized and securely handled.

## 3 RESULTS

### 3.1 Distribution of dementia cases, trends, and socio‐demographic characteristics of patients at FNPHM

**FIGURE 1:**
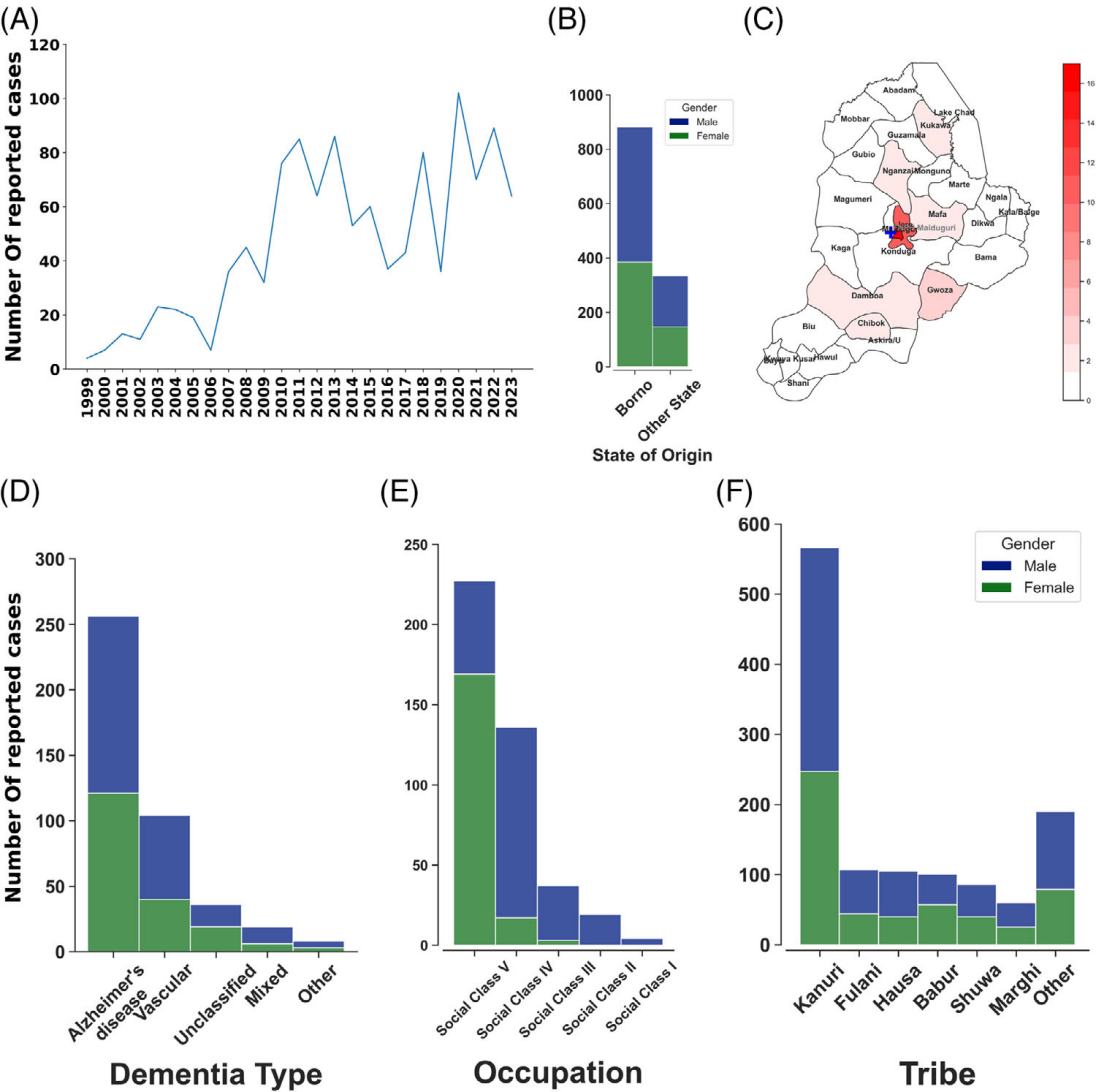
Dementia case distribution and trends at Federal Neuropsychiatry Hospital, Maiduguri (FNPHM). (A) The trend of dementia cases at the Psychogeriatric Unit of FNPHM from 1999 to 2023. (B) Distribution of patients by state of origin and gender. (C) Choropleth map of Borno State showing the number of reported dementia cases per 100,000 population among patients from Borno State. (D) Distribution of dementia types at the hospital over the last 5 years. (E) Social class distribution of patients over the last 5 years. (F) Tribal distribution of patients since the inception of the hospital.

Since the inception of FNPHM, a total of 1216 dementia cases have been reported in patients 60 years of age and older. Among these, 655 (56%) were male with a mean age ± SD of 71.4 ± 9.7 years, and 509 (43.7%) were female with a mean age ± SD of 72.7 ± 8.3 years. In the last 5 years (January 2019 to December 2023) 423 cases were recorded, with a mean age of 71.9. Of these recent cases, 234 (55.3%) were male with a mean age of 71.6 years, whereas 189 (44.7%) were female with a mean age of 72.2 years. As shown in Figure 1A, there has been an upward trend in reported dementia cases over time.

By state of origin, 857 patients (≈70%) were from Borno State, making it the most represented state (Figure 1B). Yobe State followed with 130 cases (11.2%), Adamawa State with 49 cases (4.2%), and Bauchi State with 20 cases (1.6%). Jigawa and Sokoto States reported 19 cases (1.6%) and 12 cases (1.0%), respectively. No other state had more than 10 cases. For patients from Borno State, analysis by local government areas (LGAs) (Figure 1C) showed that Maiduguri and Jere LGAs, both urban areas, had the highest number of cases, with 17 cases per 100,000 population in Maiduguri and 10 cases per 100,000 in Jere. No other LGA reported more than two cases per 100,000 population.

Over the last 5 years, Alzheimer's disease (AD) was the most frequently reported form of dementia, accounting for 60.5% of cases (Figure 1D). Vascular dementia followed at 24.5% and mixed dementia at 4.5%. A small proportion of cases were unclassified. Other forms of dementia, including dementia due to head injury, depressive pseudodementia, and frontotemporal dementia, were very uncommon.

The social class distribution of patients over the last 5 years (Figure 1E) showed that the majority, 53.7%, belonged to social class V, consisting of unemployed individuals. This was followed by social class IV, with 32.2%, including unskilled workers such as petty traders, subsistence farmers, and messengers. Social class III, comprising low‐skilled workers such as junior clerks, drivers, mechanics, and junior military and police personnel, accounted for 8.7% of cases. Social class II, representing intermediate‐skilled professionals like technicians and nurses, has fewer cases (4.5%). Cases were rare in social class I, consisting of highly skilled professionals such as doctors, lawyers, and business executives.

Most dementia cases were reported among the Kanuri tribe (Figure 1F), 46.5% of the total. The Fulani tribe followed with 8.8%, the Hausa and Babur with 8.6% and 8.3%, respectively, the Shuwa tribe with 7.1%, and the Marghi with 4.9%. Other tribes collectively accounted for 15.7% of cases.

### 3.2 Reported comorbidities and risk factors in dementia patients attending FNPHM (2019–2023)

**FIGURE 2:**
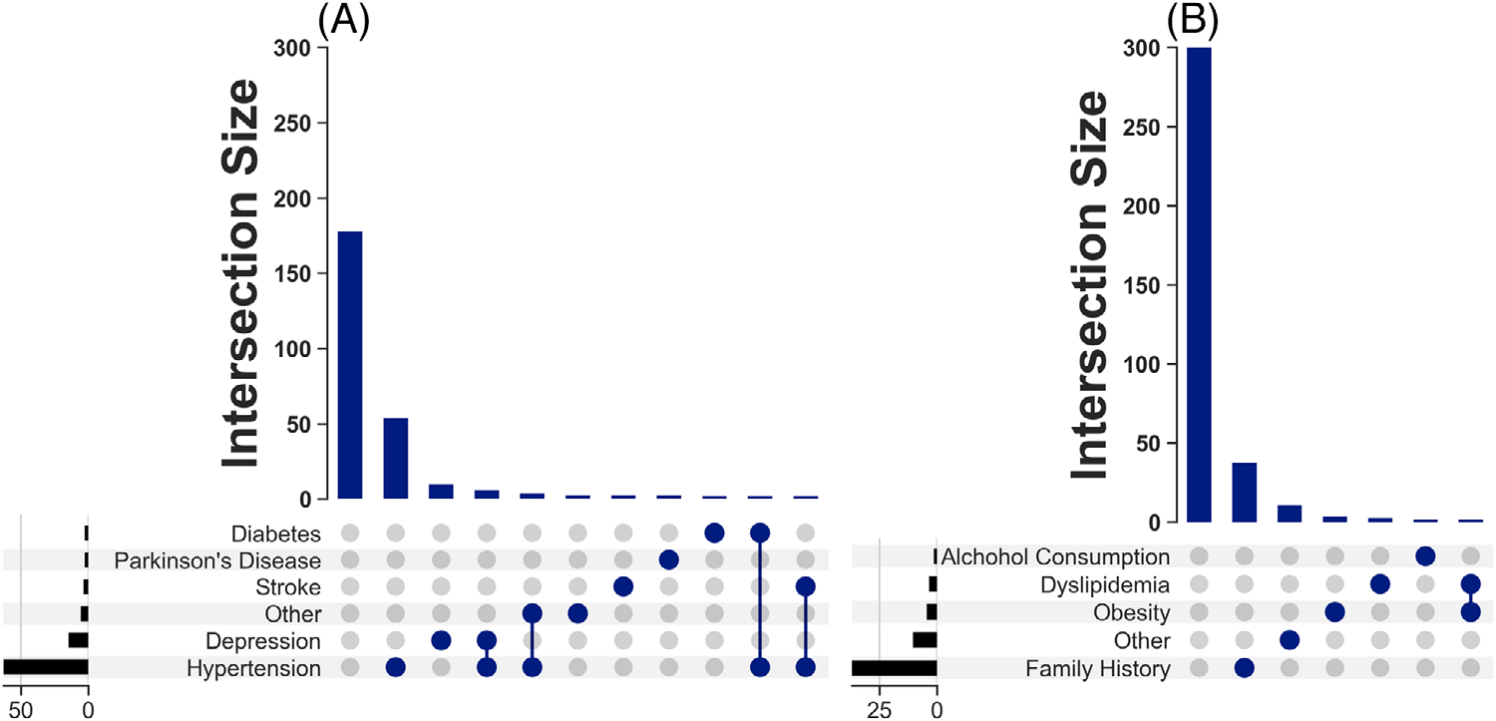
Reported comorbidities and risk factors in patients with dementia attending Federal Neuropsychiatric Hospital, Maiduguri (2019–2023). (A) Comorbidities. (B) Predisposing risk factors.

Hypertension was the most common comorbidity (Figure 2A), occurring in 41.6% of cases. The frequency of depression was 5.4%, that of stroke was 2.8%, and for diabetes, 0.7%. Parkinson's disease was observed in 0.9% of cases. Notably, 51.8% of cases had no comorbidity. Correlation analyses showed that 36.2% had hypertension and no other comorbid conditions. Hypertension combined with depression was observed in 2.4% of cases. Among the predisposing factors in patients with dementia (Figure 2B), family history was the most prevalent, reported in 8.7% of cases. Other predisposing factors were infrequent.

We analyzed comorbidities and risk factors in patients with AD, the most prevalent dementia subtype in our data set, and found that the proportions of hypertension, depression, stroke, and family history closely matched those in the overall dementia population. This similarity suggests no significant differences in comorbidity and risk factor profiles of the AD patients compared to our overall dementia population. Based on these findings, we focused on analyzing dementia management holistically rather than conducting separate analyses for AD patients.

### 3.3 Reported symptoms and investigations conducted in dementia patients attending FNPHM (2019–2023)

**FIGURE 3:**
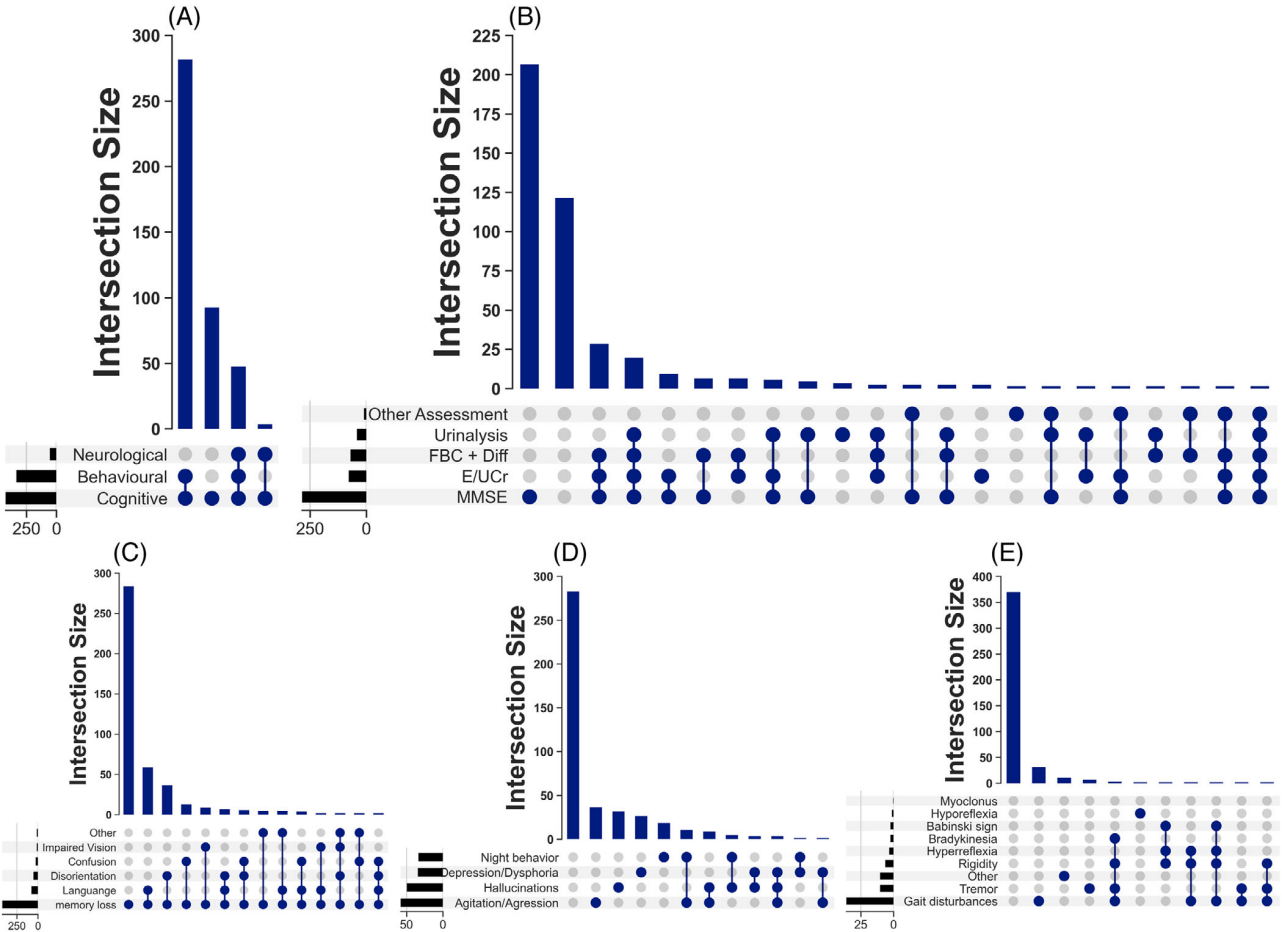
Reported symptoms and investigations conducted in dementia patients attending Federal Neuropsychiatric Hospital, Maiduguri (2019–2023). (A) Combination of reported symptoms. (B) Combination of investigations conducted. (C) Combination of reported cognitive symptoms. (D) Combination of reported behavioral symptoms. (E) Combination of reported neurological symptoms.

Among the reported symptoms (Figure 3A), cognitive symptoms were the most prevalent, occurring in all cases. Behavioral symptoms were reported in 77.5% and neurological symptoms in 11.8%. Cognitive and behavioral symptoms were noted in 66.4% of the cases. A smaller proportion, 21.8%, had only cognitive symptoms. The combination of cognitive, behavioral, and neurological symptoms was observed in 11.1%, whereas cognitive and neurological symptoms were present in 0.7%.

Memory loss was observed in all the cases (Figure 3C). Language difficulties were reported in 17.3%, disorientation in 11.6%, confusion in 5.2%, and impaired vision in 2.4% of cases. In terms of cognitive symptom combinations, 66.9% had memory impairment alone. Memory loss combined with language difficulties was observed in 13.7%, and memory loss combined with disorientation in 8.5%. The combination of memory loss and confusion was noted in 2.8%.

Of the reported behavioral symptoms (Figure 3D), agitation/aggression was the most commonly reported, occurring in 13.7%. Hallucinations were observed in 11.6%, followed by depression/dysphoria in 8.0% and night behavior disturbances in 7.8%. Notably, the majority, 66.7%, reported no behavioral symptoms. Regarding combinations of behavioral symptoms, agitation/aggression and night behavior disturbances were reported together in 2.4% of cases.

Of the reported neurological symptoms (Figure 3E), gait disturbances were the most frequently reported, observed in 8.5% of cases. Tremor was observed in 2.4%. Other neurological symptoms were far less common.

The MMSE was performed in 67.4% of cases (Figure 3B). Other routine investigations included electrolytes, urea, and creatinine assays, performed in 17.7%, and complete blood count with differential in 15.8%, and urinalysis in 9.2%. Other tests were less frequently performed.

### 3.4 Recorded treatments given to dementia patients attending FNPHM (2019‐2023)

**FIGURE 4:**
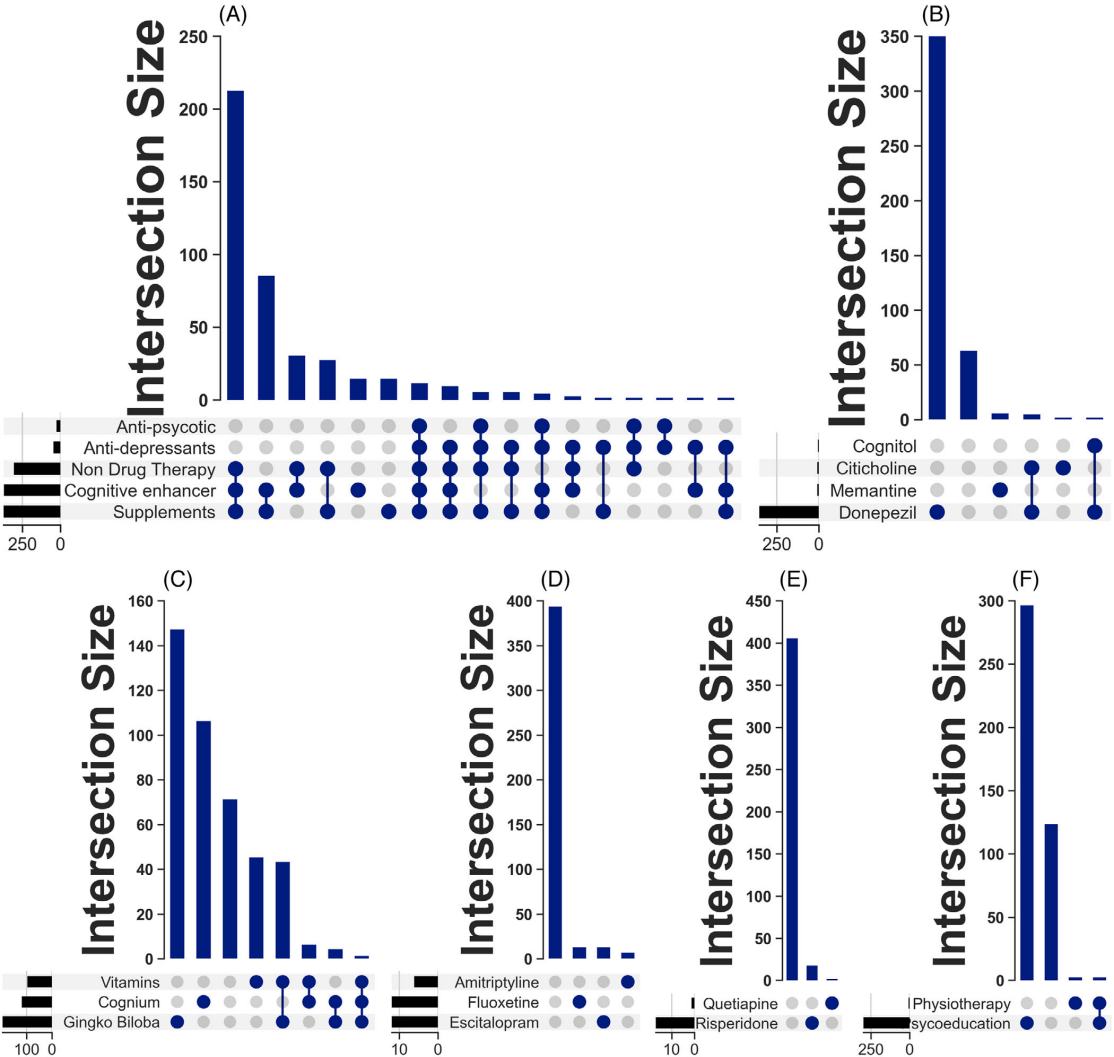
Recorded treatments given to patients with dementia attending Federal Neuropsychiatric Hospital, Maiduguri (2019–2023). (A) Combination of treatment options. (B) Cognitive enhancers administered. (C) Supplements administered. (D) Antidepressants administered. (E) Antipsychotics administered.

Supplements were the most frequently administered of the recorded treatments (Figure 4A), given in 88.4% of cases. Cognitive enhancers were used in 87.2% of cases, whereas non‐drug therapy was employed in 71.4%. Antidepressants were prescribed in 9.7% of cases, and antipsychotics in 5.2%. Regarding treatment combinations, non‐drug therapy combined with cognitive enhancers and supplements was administered in 50.1% of cases. The combination of cognitive enhancers and supplements alone was observed in 20.1%. Non‐drug therapy and cognitive enhancers together were used in 7.1%, whereas the combination of cognitive enhancers, supplements, and antidepressants was given in 0.9%.

Among the cognitive enhancers, donepezil was the most frequently administered cognitive enhancer (Figure 4B), given in 82.7% of cases. Memantine was rarely prescribed (1.2%), as was citicoline (1 case, 0.2%). The combination of donepezil and citicoline was prescribed infrequently (0.9%), and that of donepezil and vinpocetine in 1 case (0.2%). Notably, 14.7% of cases received no cognitive enhancers. Among the supplements administered (Figure 4C), gingko biloba was the most commonly used in 52.1% of cases. Silk protein hydrolysate (marketed as Cognium) was administered in 31.3% of cases, and vitamins were given in 25.4%. Combinations of supplements were given in 11.5% of cases, whereas 19.0% did not receive any supplements.

Escitalopram and fluoxetine were the most commonly prescribed antidepressants, each in 29.3% of cases (Figure 4D). Amitriptyline was prescribed in 14.6% of cases. The remaining patients did not receive any antidepressants. Risperidone was the most administered antipsychotic in 77.3% of cases. Quetiapine was used in just one case (4.5%). The remaining patients did not receive any antipsychotic medications.

Of the non‐drug therapies (Figure 4F), psychoeducation was the most frequently utilized intervention, reported in 80.1%. Physiotherapy was used in two cases (1.7%), whereas the combination of psychoeducation and physiotherapy was recorded in two cases (1.7%). In addition, 16.5% did not receive any non‐drug therapy.

A significant proportion of cases were lost to follow‐up, with 70.9% of the cases not returning for further evaluation or treatment. In contrast, 123 patients (29.1%) were confirmed to be alive at the time of the last follow‐up.

## 4 DISCUSSION

The upward trend in dementia observed here aligns with global patterns where aging populations and improved diagnostic capabilities have increased case reporting.^28^ The dips in case reporting may be linked to WHO's Mental Health Gap Action Programme (mhGAP).^29^ During WHO‐provided free treatment years, more patients likely sought care at FNPHM. Fewer patients likely visited when these interventions were unavailable. This highlights the influence of external factors on health care utilization and reporting trends.

Male dominance here contrasts with the global trend of higher female prevalence, particularly in AD.^30^ Similar female dominance has been reported in studies from Uganda, South Africa, and Lagos, Nigeria.^31^, ^32^, ^33^ However, male‐dominant trends have also been observed in other parts of sub‐Saharan Africa,^34^, ^35^ potentially due to cultural factors, and disparities in health care access between genders.^34^, ^35^ The significant representation of patients from Borno State may be attributed to FNPHM's status as a major health care provider in the region.^36^

The predominance of AD reflects local and global patterns, with AD as the most common dementia subtype.^24^, ^30^, ^31^, ^32^, ^33^, ^34^, ^37^ However, the findings contrast with a hospital‐based study conducted in Ibadan, Nigeria, between 1984 and 1989, which reported vascular dementia as the predominant subtype.^38^ Although this study had a small sample size,^38^ the results highlight the high frequency of vascular dementia in Nigeria, which was also the second most common in our data set. This substantial proportion of vascular dementia cases may be linked to the high prevalence of hypertension in Nigeria. Similar findings have been reported across Africa.^39^

Across social classes, the dominance of Social Class V (unemployed), highlights the role that socioeconomic factors play in dementia risk. Lower socioeconomic status has been linked to a higher risk of developing dementia.^34^, ^39^, ^40^, ^41^

The higher prevalence of dementia among the Kanuri tribe (46.5%) compared to other tribes may be due to their predominant representation in the population of Borno State. Although the literature on tribal variations in dementia prevalence is limited, these findings suggest the need for more research to better understand these differences.

The dominance of hypertension as the most common risk factor aligns with previous studies.^42^, ^43^ Hypertension contributes to cerebrovascular damage, which can lead to cognitive decline and dementia.^42^, ^43^ The high prevalence of hypertension in this study likely reflects broader regional, national, and continental trends, where hypertension is a common and often undiagnosed or poorly managed health issue—which exacerbates its impact on cognitive health.^44^

The relatively lower occurrence of depression, stroke, and diabetes may reflect either underdiagnosis or a genuinely lower prevalence of these conditions. Depression, for instance, is a well‐known risk factor and comorbidity in dementia, and it can mimic cognitive decline.^45^ The low reporting rate could suggest challenges in recognizing and diagnosing depression in patients with dementia, a concern highlighted in studies from other resource‐constrained settings.^46^ Similarly, the small number of cases involving stroke and diabetes—both recognized risk factors for dementia—may point to limitations in screening or reporting practices, which is often an issue in low‐resource environments.^47^, ^48^

The low report on comorbidities raises concerns about the completeness of medical records and/or the thoroughness of patient evaluations. It is possible that some comorbidities were not identified due to limitations in diagnostic capabilities or the prioritization of dementia management over the identification of other conditions. Another contributing factor could be the overwhelming patient volume at the hospital or across the country, which may restrict doctors' ability to conduct thorough assessments. In Nigeria, where the doctor‐to‐patient ratio is critically low, health care professionals are often overburdened with large caseloads.^49^ There is currently about 1 neurologist per 2 million people in Nigeria.^50^

The prominence of family history as a risk factor for AD has long been established in other populations. However, the overall low reporting of other predisposing factors can be attributed to a lack of detailed medical histories or challenges in collecting accurate data. This is typical of studies in low‐resource settings, where these factors are often underexplored due to limited patient interaction, or cultural sensitivities.^51^

The reporting of memory loss in all cases is consistent with findings globally highlighting memory loss as the most prominent early symptom of dementia.^52^, ^53^, ^54^, ^55^, ^56^, ^57^ The presence of language difficulties, disorientation, and confusion in a smaller proportion of cases aligns with global observations. For example, language difficulties are often associated with more advanced stages of dementia.^58^, ^59^

The high prevalence of behavioral symptoms is also consistent with the literature on dementia. The BPSD, including agitation, aggression, and hallucinations, are well documented and often challenging to manage.^60^, ^61^, ^62^ Other studies demonstrate that these symptoms not only cause significant distress to patients and caregivers but are also predictors of institutionalization.^6^, ^63^, ^64^ The fact that a significant portion of patients reported agitation/aggression and hallucinations is typical of the BPSD.

The lower prevalence of neurological symptoms is in line with other studies where these symptoms are typically less common in the early stages of dementia but may become more pronounced as the disease progresses. These are often indicative of more extensive neurodegeneration or advanced disease in conditions like Parkinson's disease dementia.^65^

The observed combinations of symptoms align with the previous studies, which emphasize the fact that patients often present with symptoms that span cognitive, behavioral, and neurological domains, further complicating diagnosis and management.^66^, ^67^, ^68^

The lack of detailed data on investigations conducted at FNPHM reflects the resource constraints typical of many sub‐Saharan African health care settings.^39^ The limited access to advanced diagnostic tools like neuroimaging and biomarker assays can lead to a reliance on clinical symptoms for diagnosis, potentially contributing to underdiagnosis or misdiagnosis of dementia subtypes. This might also explain the lower reporting of certain symptoms, which may require more sophisticated clinical approaches to be accurately identified.^39^, ^69^

The frequent administration of cognitive enhancers and supplements at FNPHM reflects global trends in dementia care, where these interventions are commonly used to manage symptoms and improve quality of life.^34^, ^35^, ^70^ The predominant use of donepezil among cognitive enhancers aligns with its established efficacy in improving cognitive function in AD.^35^, ^71^ The limited use of memantine, despite its recognized benefits in moderate to severe AD, is due to resource constraints at FNPHM, particularly its cost and limited availability in a low‐resource setting. When available, its use may be restricted to cases where a patient's diagnosis specifically warrants it. Studies show that ginkgo biloba is often utilized for its neuroprotective properties, which can support cognitive function in patients with dementia,^72^ supporting their extensive use at FNPHM.

The conservative use of antidepressants and antipsychotics in this study aligns with the caution recommended in the literature regarding their use in the management of BPSD. Antidepressants such as escitalopram and fluoxetine are prescribed to manage depressive symptoms, which are common in dementia, but their benefits must be carefully weighed against potential adverse effects, particularly in older adult patients.^73^ This approach is consistent with findings that highlight the need for careful consideration of the risks and benefits of psychotropic medications in dementia care.

The limited use of antipsychotics, with risperidone being the most administered, reflects best practices that advise minimizing the use of these drugs due to their association with increased mortality and cardiovascular adverse events in patients with dementia.^73^, ^74^ Current guidelines emphasize non‐drug approaches as the first line of treatment for BPSD, reserving antipsychotics for severe cases where other interventions have failed. The high prevalence of non‐drug therapies, particularly psychoeducation, supports the recommendations in the literature, which highlight the importance of non‐pharmacological interventions. Several studies have highlighted the role of such therapies in managing BPSD, which are often inadequately addressed by medication alone.^67^, ^75^ The reliance on psychoeducation at FNPHM reflects an understanding of the value of patient and caregiver education in managing dementia, particularly in settings where access to advanced pharmacological treatments may be limited.

The combination of non‐drug therapy, cognitive enhancers, and supplements in more than half of the cases at FNPHM aligns with an approach to dementia care that integrates multiple modalities to address the complex needs of patients. For example, some studies have emphasized the potential synergistic effects of combining different synthetic and natural compound classes to enhance therapeutic efficacy.^76^

The significant loss to follow‐up among patients with dementia reflects cultural attitudes, where many Nigerians seek medical care only when symptomatic and often discontinue follow‐up once they feel better. In addition, incomplete reporting of deaths confounds the tracking of patient outcomes. These factors, common in LMICs, hinder the ability to assess the effectiveness of treatments. The resource constraints in Borno State worsened by the Boko Haram conflict have severely impacted clinical care in the region.^77^ Limited health care infrastructure, medical supplies, and reduced access to specialized services lead to delayed diagnosis and poor follow‐up, thereby hindering comprehensive dementia care in the region.

Overall, our study provides valuable insights into dementia trends and management in a resource‐constrained setting, despite inherent limitations, including reliance on retrospective clinical records, limited diagnostic tools, and patient loss to follow‐up. Further research is essential to overcome these challenges and improve dementia diagnosis, care, and long‐term outcomes in underserved regions.

### 4.1 Recommendations

To improve dementia diagnosis and care in resource‐constrained settings, we recommend equipping health care facilities with advanced diagnostic tools, including biomarker and imaging analysis equipment, and establishing well‐characterized cohorts to define biomarker reference parameters and better understand disease progression. Educating patients and caregivers on continuous care, supported by reminders, home visits, or telehealth, is essential to ensure regular follow‐up. Strengthening communication between facilities and families for accurate documentation and implementing community‐based interventions to address cultural attitudes and reduce dementia stigma are equally critical. Partnering with local leaders can foster a proactive approach to care. Allocating resources to train health care providers in non‐drug therapies and comorbidity management will enhance care quality. Finally, prioritizing research on treatment effectiveness in low‐resource settings and improving data collection methods will better track outcomes, efficacy, and mortality rates.

## AUTHOR CONTRIBUTIONS

The study was conceived, organized and supervised by Mahmoud Bukar Maina, Ibrahim Abdu Wakawa, and Chiadi U. Onyike. Data curation was done by Umar Baba Musami, Suleiman Hamidu Kwairanga, Placidus Nwankuba Ogualili, Mohammed Yusuf Mahmood, Muhammad Abba Fugu, Mohammed Mala Gimba, Muktar Mohammed Allamin, Zaharadeen Umar Abbas, Muhammad Kawu Sunkani, Zainab Bukar Yaganami, Fatima Mustapha Kadau, Nasir Muhammad Sani, Peter Danmallam and Luka Nanjul managed by Mahmoud Bukar Maina, and Ibrahim Abdu Wakawa. Data analysis was done by Suleiman Hamidu Kwairanga. The article was written with contributions from all authors. Methodology and study design were guided by Babagana Kundi Machina, Baba Waru Goni, Larema Babazau, Zaid Muhammad, Suleiman Hamidu Kwairanga, Chinedu Udeh‐Momoh, and Thomas K. Karikari. Mahmoud Bukar Maina, Ibrahim Abdu Wakawa, Chiadi U. Onyike, Chinedu Udeh‐Momoh, Celeste M. Karch and Thomas K. Karikari provided expert guidance and facilitated discussions throughout the project.

## CONFLICT OF INTEREST STATEMENT

Dr. Mahmoud Bukar Maina received funding for this work from the Rainwater Charitable Foundation and the Alzheimer's Association. All other authors declare no conflicts of interest related to the content of this manuscript. Author disclosures are available in the Supporting Information.

## CONSENT STATEMENT

Ethical approval for this study was obtained from the Health Research and Ethics Committee of the University of Maiduguri Teaching Hospital. Informed consent was not required, as anonymized patient data were used.

## Supporting information



Supporting Information
